# RETRACTED: Covarelli et al. Retinoic Acid Induced 1 and Smith–Magenis Syndrome: From Genetics to Biology and Possible Therapeutic Strategies. *Int. J. Mol. Sci.* 2025, *26*, 6667

**DOI:** 10.3390/ijms27209366

**Published:** 2026-10-21

**Authors:** Jasmine Covarelli, Elisa Vinciarelli, Alessandra Mirarchi, Paolo Prontera, Cataldo Arcuri

**Affiliations:** 1 Department of Medicine and Surgery, University of Perugia, Piazza L. Severi 1, 06132 Perugia, Italy; cova97@gmail.com (J.C.); alessandra.mirarchi@unipg.it (A.M.); 2 Medical Genetics and Rare Disease Unit, Maternal-Infantile Department, S. Maria della Misericordia Hospital, 06132 Perugia, Italy; elisa.vinciarelli@edu.unife.it (E.V.); paolo.prontera@ospedale.perugia.it (P.P.); 3 Department of Translational Medicine and for Romagna, University of Ferrara, Via L. Borsari 46, 44121 Brescia, Italy

The journal retracts the article titled, “Retinoic Acid Induced 1 and Smith–Magenis Syndrome: From Genetics to Biology and Possible Therapeutic Strategies” [1], cited above.

Following publication, concerns were raised by a reader regarding the identification of the RAI1 gene analyzed in this study [1].

In accordance with the journal’s standard procedures, an investigation was conducted by the Editorial Office and the Editorial Board, which confirmed that the gene described as RAI1 was incorrectly identified, resulting in an inaccurate interpretation of the published literature discussed in this review [1]. Furthermore, the Editorial Board determined that the extent of the necessary revisions exceeded what could be addressed through a standard correction. The author collaborated with the investigation and acknowledged the error.

As a result, the Editorial Board decided to retract this article in line with MDPI policy on retraction.

This retraction was approved by the Editor-in-Chief of *International Journal of Molecular Sciences*.

The authors have been informed of this retraction but did not provide a comment on this decision.
